# Routinized violence: examining long-term residential care workers’ perspectives on involuntary treatment

**DOI:** 10.1093/geront/gnaf215

**Published:** 2025-09-19

**Authors:** MacGregor Goodman, Laura M Funk, Rachel V Herron, Sheila Novek

**Affiliations:** School of Social Work, York University, Toronto, Ontario, Canada; Department of Sociology and Criminology, University of Manitoba, Winnipeg, Manitoba, Canada; Department of Geography and Environment, Brandon University, Brandon, Manitoba, Canada; Department of Psychiatric Nursing, Brandon University, Winnipeg, Manitoba, Canada

**Keywords:** Dementia, Restraint, Involuntary treatment, Qualitative

## Abstract

**Background and Objectives:**

In long-term residential care (LTRC), sometimes workers provide treatment that residents refuse or resist, which can cause harm to both workers and residents. In this analysis, we explored how and when workers provide involuntary treatment, when they accept or see this practice as necessary and when they reject this practice.

**Research Design and Methods:**

Following a qualitative research design, data were collected through interviews with nurses, health care aides, recreation, and housekeeping staff in two Canadian provinces and observations in two LTRC facilities in the province of Manitoba. Using an interpretive coding approach and guided by Foucauldian concepts of power and structural violence, we examined descriptions of violent situations and everyday interactions with a particular focus on involuntary treatment.

**Results:**

Beliefs about the potential for physical harm toward workers influenced the perceived acceptability, or rejection, of involuntary treatment. However, workers often expressed ambivalence about the acceptability of certain practices (e.g., using multiple workers to hold down a resident to provide personal care). The potential for worker injury and risk of being reprimanded were frequently identified by workers as shaping their decisions about whether to proceed with treatment to which the resident had not consented. At times, workers also expressed obligation to provide involuntary treatment for biomedical reasons, or because there seemed to be no good alternative.

**Discussion and Implications:**

Workers’ narratives about involuntary treatment reflect a lack of interpersonal and organizational safety that undermines the autonomy and dignity of those for whom they provide care.

In recent decades, gerontologists have examined various forms of violence—acts that may cause social, emotional, sexual, and/or physical harms—in long-term residential care (LTRC) (Conti et al, 2022; Pillemer et al., 2024; Song et al., 2023). LTRC workers experience high rates of violence (Banerjee et al., 2015; Braedley et al., 2018; Brophy et al., 2018), as do residents, particularly those who live with dementia (Mileski et al., 2019; Steele et al., 2023). Studies have identified correlations between resident aggression toward workers and physical abuse and neglect by workers toward residents (Botngård et al., 2021; Cooper et al., 2018; Hirt et al., 2022). For example, when staff provide treatment residents do not consent to, residents may respond aggressively and in turn staff may become more forceful in their efforts to provide treatment. Consequently, LTRC can reinforce relationships of mutual harm for residents and workers, often in subtle ways that undermine dignity, autonomy, and even violate human rights (de Boer et al., 2019; Steele & Swaffer, 2022). Attempts to cope with harms and stressors associated with this unsafe environment may reinforce destructive interpersonal dynamics between residents and workers and normalize violence within their interactions (Funk et al., 2021). **To address these mutual harms, gerontologists must continue to question and problematize manifestations of routinized violence nested within care practices, including the use of restraints, coercion, and involuntary treatment**.

To address less overt forms of coercion within LTRC, in 2020, the Netherlands introduced an act prohibiting “involuntary care,” which refers to care that residents do not consent to or resist (de Boer et al., 2019; Willems et al., 2023). Although workers’ lack of time, experience, and awareness of the concept of involuntary care hampered the acts’ implementation in that country (Willems et al., 2023), the act nonetheless presents an important step in naming, describing, and regulating routinized violence toward LTRC residents. In Canada, where this study took place, the term involuntary treatment is generally used to refer to medical interventions undertaken against a person’s will. Decisions about involuntary treatment are governed by mental health regulations in each province and therefore operate outside of the LTRC sector. **To our knowledge, there is no research in Canada on involuntary treatment in LTRC.** 

Building on scholars such as Kelly (2017), who emphasize the ways that violence is intertwined with care provision (Kelly, 2017), we use an interpretive approach to explore how workers describe involuntary treatment in their everyday interactions and violent situations. **We argue that involuntary treatment is not care; it’s part of a larger system of routinized violence perpetuated by workers who feel obligated to protect themselves and do their jobs and by residents who resist and refuse their treatment.** In the section that follows, we outline international policies aimed at addressing forms of violence and coercion as well as theories of care, control, and resistance that inform our work.

## Involuntary treatment, care, and resistance in LTRC

Various concepts have been used to examine the ethicality of treatment provision, including “involuntary care,” “non-consensual care,” and “involuntary treatment” ­(Moermans et al., 2022; Willems et al., 2023). Such terms typically refer to the measures that limit a person’s control over their life, when the person does not consent to or resists such measures, including physical and chemical restraints, restricting access to mobility aids, forcing hygiene, or locking a person in their room (Moermans et al., 2022; Willems et al., 2023). **In this article, we use the concept of “involuntary treatment” rather than coercive or involuntary “care” in part to trouble the notion that coercive practices can be considered care at all.** 

Some practices that restrict autonomy and cause harm to residents, such as the use of physical and chemical restraints, are regulated through policy directions and practice requirements. For example, in North America, Australia, and parts of Europe, the use of chemical and physical restraints is only condoned in policy as a last resort (i.e., least restraint policies), after other options have been exhausted (Hirt et al., 2022). However, restraints are still frequently overused (Canadian Institute for Health Information, 2021; Hamers, 2017; Lee et al., 2021). Barriers to implementing alternative practices include a lack of policy clarity regarding what constitutes a restraint, concerns about resident safety, insufficient staffing and resources, and lack of decision-making supports (Pu & Moyle, 2022). Policies that primarily emphasize staff responsibility for violence prevention can obscure structural and organizational factors shaping harmful practices (Novek et al., 2022), as well as more subtle restraints and deceptive practices such as hiding medications in food and drink (Robins et al., 2021). Furthermore, staff are often left to navigate conflicting policy directions, documentation requirements, and potential disciplinary measures for failing to comply with regulations. Lastly, existing policies can normalize or justify restraints and other forms of routinized violence by reproducing stigmatizing and dehumanizing narratives about the capacity and behavior of people living with dementia, in part through one-dimensional characterizations of vulnerability and dangerousness (Chelberg, 2023), and through systematic exclusion from policy development processes (Chandler et al., 2018).

In addition to policy regulation, person-centered and relational approaches to care have promoted a cultural shift in care provision. Person-centered care is a philosophy and dementia care practice which involves recognizing residents’ personhood and sense of self (Kitwood & Bredin, 1992). In developing this approach, Kitwood (1997) used a theoretical perspective that has been conceptualized as Foucauldian (Davis, 2004) to analyze institutional care for individuals living with dementia. He rejected the normalization of dehumanizing and abusive “care” for people with dementia and the biomedical perspective that all disease has an organic etiology, arguing that pervasive medicalization leaves no room for personhood (Kitwood, 1997).

According to social theorist Michel Foucault, resistance is both an exercise of and a response to power: it is also its natural accompaniment (Foucault, 1995). Conceptualizations of power relations as static and hierarchical can limit analyses, particularly within examinations of multidirectional violence and harm. Foucauldian theory is useful, then, in framing the complex power dynamics between workers and residents that manifest in an institutional setting where neither are particularly empowered (Suijker, 2023). For instance, previous research has illuminated epistemological violence within LTRC that devalues and underutilizes care workers’ knowledge and expertise, by removing the opportunity for the compassionate and relational care that workers describe wanting to provide (Banerjee et al., 2015; Kontos et al., 2017). The concept of epistemological violence can also be applied to the treatment of residents, where residents’ attempts to communicate their lack of consent to treatment are discounted and treated as invalid. Epistemological violence undermines both workers’ and residents’ by discrediting their capacity to *know*, whether how to do their job or to decide what they want done to their body (LeBlanc & Kinsella, 2016; Spivak, 2004). The pathologization of residents’ resistance can reaffirm a kind of institutional power in response to residents’ assertion of their own autonomy. When residents respond to this lack of regard, any agitation or aggression may be attributed to the individual’s dementia rather than trying to understand why they are agitated (Braaten & Malmedal, 2017). Altogether, this exemplifies how the medicalization and carcerality of LTRC operate through what Foucauldian scholars have termed “technologies of domination” (Mortenson et al., 2015).

The prioritization of medical and physical needs in LTRC further reflects a “medical gaze” that permeates institutions of care, creating a culture that devalues relational care (Foucault, 1978). This can enable violent interactions; in some settings, aides are expected to behave in a manner that is dehumanizing and abusive to prioritize care oriented around medical and physical needs (Kelly, 2017). Intimate, bodily treatment performed without consent may be expected of workers, even though this could be experienced as an assault by residents, causing physical and mental harm, and may not be seen as acceptable outside of the institution. In the context of heavy workloads, insufficient time, insufficient staffing, and task-oriented care delivery, care workers may feel compelled to provide care despite experiencing significant distress when faced with how these conditions negatively impact residents (Braedley et al., 2018; Reynolds et al., 2022). Indeed, respecting residents’ refusal of care could be perceived as inadequately fulfilling the expectations and norms of their role (e.g., as neglect).

Building on recent research on involuntary treatment (de Boer et al., 2019; Willems et al., 2023), we explicate how both workers and residents may be attempting to protect themselves from harm while living and working in institutions that have failed to protect them. We develop this analysis through examining violent situations that appear to occur because of involuntary treatment from staff and situations in which staff endorse the use of involuntary treatment to manage violent situations. In these cases, we ask how and when workers provide involuntary treatment, when they accept or see this practice as necessary, and when they reject this practice. In doing so, we contribute to critical gerontological perspectives on how structural policies and conditions create an environment that perpetuates mutual harm rather than care (Banerjee et al., 2012; Banerjee et al., 2015). We further identify this as “structural violence,” which is a concept that describes how policies and practices within organizations can stifle the well-being of the people who live and work in those institutions (Banerjee et al., 2012; Herron & Wrathall, 2018). In this regard, systems, policies, and practices that normalize and enable harmful behavior can be understood as violent in and of themselves.

## Research design

To explore the forms of involuntary treatment workers describe, accept, or reject and the contexts in which they do so, we draw on data from a larger study of violence in LTRC (Herron et al., 2021). The broader study sought to examine how the physical, social, and cultural features of LTRC environments influence violent situations, including understanding regional and organizational policies and individual practices to prevent or manage violent situations (Novek et al., 2022). Both policies and practices around subtle or more overt restraints, involuntary treatment, and coercion emerged early in data collection for this study prompting this analysis. Data sources for this analysis included 29 telephone and web-based interviews with LTRC workers in Manitoba and Nova Scotia, Canada conducted from 2021 to 2023, alongside intensive observational data and 10 key informant interviews collected from two Manitoba long-term care facilities over the course of 2 weeks in each site.

### Interviews

After obtaining ethics approval from affiliated university research ethics boards and regional health authorities, a purposive sample of LTRC workers was invited to participate through material shared by professional associations and non-profit organizations (e.g., professional associations for nurses, healthcare worker unions, and long-term care organizations) and through institutional social media accounts of the research team. Interested participants were encouraged to complete an online screening survey. Those who reported experiencing some form of violence at work and wished to participate in an interview were contacted by the team to arrange an interview by phone or video call. In total, 29 staff participated in these initial interviews; most (*n* = 26) identified as women; were White (*n* = 12); and almost half were 50 years of age and older; 18 were nurses, six were healthcare aides, two were recreational staff, and three provided housekeeping services.

All interview participants provided written informed consent prior to the interview, which followed a semi-structured guide. Participants were asked to describe their role, training, and interactions with residents, family, and workers; their experiences of violent situations; and violence prevention strategies and policies. While describing violent situations, participants detailed times and places where they used subtle or more overt restraints and provided involuntary or coercive treatment. On average, interviews lasted approximately 80 min. All interviews were digitally recorded and transcribed verbatim to enhance confirmability and credibility.

### Observation

Following remote data collection, two LTRC facilities (referred to here as Facility A and Facility B) were selected for observational research in discussion with a knowledge user advisory committee. The committee selected two sites by group consensus, based on information about various site selection criteria they identified (e.g., ownership, urban/rural, large/small, new build/household models, religious and cultural affiliations, and different philosophies of care) and what they knew about promising initiatives and policies implemented in different facilities to address violence. The principal investigator invited these sites and subsequently discussed the study with each facility’s directors and family and resident councils.

Both facilities were publicly funded non-profit organizations in small towns; Facility A was a larger newer building, and Facility B was a smaller older building. To preserve the confidentiality of the sites and individuals working there, we have only included generalized details about the facilities. Notably, Facility A had implemented a policy prohibiting what they called “forcing care.” Observations at Facility A were in their behavioral unit, where residents were placed due to past violent incidents. All residents in Facility A were older adults with dementia or another cognitive degenerative condition. In Facility A, two residents, 15 workers, and one family member consented to observation. In Facility B, 14 residents, 16 workers, and one family member consented to observation. The research team were able to observe the general area of Facility B, where there was greater variation in residents’ ability to provide verbal consent. All participants have been given pseudonyms to protect their confidentiality, and all identifying information has been redacted.

At the outset of the observational research, three key informant interviews were conducted with workers and leadership in Facility A, and seven were conducted in Facility B to understand the study sites, organizational culture, and policy context and their impact on care provision. Data about the age, gender, and race of these participants was not collected. With written informed consent from workers and consent or assent from residents and family carers, and after a one-day trial to build rapport, refine the observation guide, and obtain the organizations’ feedback, the observations commenced. Rather than focus exclusively on violent acts, the research team followed a standardized observation guide focused on documenting the social, organizational, and physical environment and situations that might be characterized as more subtle manifestations of violence or practices and interactions that might contribute to violence. To support data triangulation within and across small groups, five researchers worked in groups of two observing for a 2-hr shift that took place between 7:30 a.m. and 7:30 p.m. After each shift, a new team would observe while the first team refined their notes. At the end of each day, the whole team debriefed, sharing emergent themes and reflecting on interactions with participants as well as ethical considerations. The combination of a standardized observation guide, multiple observers documenting and triangulating their notes during each time period, and continued team debriefing to critically reflect on the research process was used to ensure consistency, comparability, and rigor throughout the observations (Armstrong & Lowndes, 2018). Importantly, during our team debriefings, we began to explore more nuanced forms of manipulation and coercion that workers employ while negotiating care.

In the first stage of analysis, textual data from the interviews and observations were analyzed separately using an inductive coding process that involved two researchers reviewing all text to develop preliminary codes that were presented to the larger research team for analytic discussion and clarification (Saldana, 2021). While these two data sources are very different, this concurrent inductive approach was critical to understanding both what care workers say directly about involuntary treatment and how they act in the particular situations the research team had the opportunity to observe. Looking at the initial code report, 120 references to forced and non-forced care were found in 36 sources. In a second phase of focused coding, the first author focused on descriptions of what the team initially termed “forced care,” and developed sub-codes to describe different actions in the textual data from both interviews and observations (e.g., restraining a resident using a Broda chair, holding a resident’s hands down, multiple workers holding a resident down, and providing care without permission or communication). Interpretive codes were developed, focusing on staff explanations for involuntary treatment (e.g., mitigating risk to the worker, preventing violence, fear of reprimand, and providing necessary care). Throughout this analytic process, the first author applied Foucauldian concepts of power by writing interpretive memos about the actions and explanations observed in the data. For example, data excerpts that referred to multiple workers holding people down were compared to explore the power dynamics and explanations across cases. We then synthesized our analysis of workers’ actions and explanations into four interpretive themes that illuminate the complex meanings and contextual constraints shaping the use (or non-use) of force in this setting; these are presented below after an introductory overview of the findings.

## Findings

In the majority of interviews, workers expressed that they often felt unsafe in their workplace, whether due to the risks of physical injury, judgment and reprimand from supervisors and residents’ family members, and/or a lack of security in their employment. A sense of disempowerment infused workers’ explanations of the contexts of their decisions around care provision. For instance, workers expressed feeling pressured (externally, internally, or both) to provide involuntary treatment despite the risk of experiencing violence (as illustrated below). The language that workers used to describe coercion in care sometimes revealed their attempts to protect themselves from harm, even as these efforts were to the detriment of residents. Participants mentioned several coercive tactics, most often manual forms of restraint, like holding residents’ hands down while providing treatment (and involving multiple staff members in doing so), and chemical restraint, but also more subtle forms of coercion, including misinforming residents and hiding medication in food and drink. These forms of involuntary treatment were described by workers not only as institutionally condoned, but as expected and necessary. In determining what shapes workers’ own acceptance or rejection of involuntary treatment, we identified that the prevention of harm to workers was often (understandably) a dominant consideration, although this was not necessarily equivocal. We also found that involuntary treatment was often justified by workers who stressed the importance of bodily care and hygiene over residents’ consent or lack thereof. Workers identified several dilemmas and contradictions in care provision, as well as challenges among workers when determining how to provide care.

The following four themes highlight how workers managed and responded to four concerns: (1) preventing physical injury to staff, (2) providing care efficiently, (3) preventing judgment and reprimand, (4) preventing and navigating moral distress and similar dilemmas in care.

### Concern for preventing worker physical injury in narratives and decisions about providing (or not providing) involuntary treatment

Participants frequently described an everyday practice of purposefully deploying teams of multiple workers in situations where physical force (restraint) was used on a resident when providing forms of personal care. This was conceptualized as involuntary treatment. Many participants described these practices as necessary and expected of them, to provide care without experiencing physical injury, often while grappling with complex, even contradictory, internal experiences of these norms:

*Now, if you’re on the unit that needs four or five people in each room, you kind of work as a team. That’s the good thing about it is like everybody’s been there for a long time, so everybody knows the roles that they’re kind of an expert in it, especially the healthcare aides. So, we work really well, and everybody knows like okay, you’re going to be washing, we’re going to be holding their hands so they don’t punch or kick or any of that sort of stuff.* **(Ella, Nurse)** 

Ella expressed in her interview that she believed there was insufficient time to engage in less forceful care practices, though she wished she had time. Several other workers expressed their disapproval of this practice as well, and empathized with how frightening it could be for a resident to have three or more workers in a room, forcing bodily care on them. One participant in an outreach role (Ashley) described reviewing violent incident reports they had received from other facilities, saying:

*I’ll read through the notes… “Two people went in to provide care because person was fighting, and we have to call in the third person and the fourth person.” And so, then I’m imagining this small room with this scared person with dementia, who’s got five staff holding them down, washing them, changing them, and my heart breaks, and I think “how traumatic for this person.”* **(Ashley, Nurse, Facility A)** 

Some other participants reflected a kind of ambivalence or moral distress, for instance, in reflecting that feeling “ganged up on” by workers could exacerbate resident aggression, while nonetheless maintaining that the presence of more workers for physical restraint (holding) still prevented injury should a resident respond aggressively.

Likely reflecting the inclusion of one facility with a non-forced care policy (Goodman et al., 2025) in our dataset, we identified accounts that presented an alternate perspective on forcing care as an injury prevention strategy. Specifically, some participants went beyond disapproving of involuntary treatment as harmful or frightening (as above), and highlighted their understandings of how the practice can lead to responsive behaviors in residents. Barb, who worked in a management position in facility A, explained how they developed their non-forced care policy, and what inspired its development:

*[Resident] was our catalyst, as I said. He was particularly difficult and challenging. Many people were off with injuries, we were terrified that he would hurt the smaller residents, he was very, very cognitively- like profoundly cognitively impaired. […] Initially, he had always been particularly challenging. But, I think that staff, not intentionally, created these really responsive behaviors, by holding him down and restraining him.* **(Barb, Nurse)** 

Barb believed that their restraint practice had “created” responsive behaviors in a resident who frequently used violence during care provision. Institutional norms had been established, then repeatedly reinforced and reaffirmed each time workers provided routine care, and by an identity label characterizing this resident as challenging, violent, and dangerous.

Participants interviewed from Facility A (which had shifted their care approach) thus most clearly conceptualized involuntary treatment as ethically wrong, and requiring a change in practice, in the context of an increasingly violent environment that was seriously harming workers. It was also notable that although the managerial key informant expressed critical reflection on past organizational practices and accountability for harms, the actual catalyst for organizational change was the harm that the resident was causing to workers, rather than the harm caused to residents by using physical force when attempting to provide personal care. Later in the interview, Barb also noted organizational concerns about workplace compensation as in part influencing a broader policy and practice shift.

### Concern for efficient care provision in a constrained context: Narratives and decisions about coercion without seeking consent

Further demonstrating the predominance of worker physical injury concerns (alongside narrow interpretations of force), many participants expressed support for other, more subtle forms of involuntary treatment, such as coercion without seeking consent, when providing care for residents who did *not* have a history of violent behavior or aggression (i.e., when there is less risk of injury). For instance, Ashley explained:

*When we’re dealing with violence, some staff, I think have- and again, especially as they get to know, a resident can really get a feel when that person is escalating, and there’s risk that they are going to lash out or they know, this is sort of their tendency that they’ll lash out. Whereas other people where they feel like, ‘yeah, this person is very anxious, but I* ***know they don’t have a history of hitting out. I feel like I can push a little bit harder*** *without being disrespectful to the person … without being at risk myself.’ (researcher emphasis)*

Ashley’s description of care practices was affirmed in the researcher’s observational notes:

*Two aides came over to [resident], put toileting products on his walker, and physically lifted [resident] up to stand, saying ‘let’s go’. They got [resident] to hold his walker and helped walk him to what I think was his room. [Resident] made verbal exclamations as soon as the aides came to touch him and did not sound happy but did not put up more of a fight, walking along with them. This was an interesting observation as the aides did use force to lift [resident] and did not give him warning they were doing so; however, [resident] did not appear distressed after the initial outburst, following along with them. We wondered if these aides, who were both full-time on the special care units and knew the resident presumably well, knew how far they could push and what was okay versus what was true distress, or if they simply did not care.* **(Facility A)** 

Although these more subtle coercive practices differ from engaging multiple workers to hold down residents’ body parts to perform care, the underlying justification likewise reflects a prioritization of workers’ and or organizational needs (in this case, to provide care quickly) rather than on reducing the anxiety and psychological harm residents may be experiencing.

In the next section, the predominance of workers’ and/or organizational concerns about other forms of harm for workers (organizational, professional, or family reprimand) reappears in workers’ narratives, complicating their decisions about involuntary treatment.

### Preventing reprimand and complaint: Narratives and decisions about restraint use and forcing care

In addition to concerns about the risk of violence and injury in their workplace, workers were also very cognizant of the risk of disciplinary action while navigating consent and coercion in care provision, especially in a context in which the use of some forms of restraint was highly prohibited and/or regulated. This had important implications for how they interpreted the use of restraint. For instance:

*And I’m the only RN in the building. I’ll walk onto the floor and find somebody in a wheelchair, a Broda^1^ with the seatbelt on, and I’m like, ‘What the hell was this about?* ***Nobody told me and I’m the one that’s supposed to make that decision****. What’s the person doing in that?’ Right? But it’s, ‘That’s okay, we’ve done it this way forever.’ ‘Well, no.* ***When I’m on, it’s my license on the line when we do that.*** *Like what happened, that you decided you should put them in a Broda^1^ with restraint.’ (researcher emphasis)* **(Carol, Nurse)** 

Carol retells a situation where an aide had applied a restraint to a resident, which would prevent them from getting up from their wheelchair. In explaining this, Carol rejects the use of physical restraint because of the risk to her licensure/professional liability; however, she says little about the risk of harm to the resident, or the ethicality of restricting a person’s range of movement. This reflects a context in which some LTRC workers (perhaps especially nurses who hold professional accountability) may feel a lack of security or threat of being sanctioned in their employment due to difficulty navigating policies around consent and restraint, before considering the moral or ethical challenges among workers when determining how to provide care.

Deanna detailed her active attention to concerns about preventing both injury and disciplinary action while providing treatment to residents who are actively resisting:

*So five of us go in to do these really combative ones, and I’m just like, ‘You can’t restrain them because that’s against the rules.’ I said, ‘You just protect yourself, protect your face. And, if it’s too much, […] he can sleep in a T-shirt, if he’s that combative, it’s not worth the broken nose.’ […] ‘Don’t restrain, don’t put pressure. Hold her, you can hold her hand, like hold her hand like if you’re holding hands, and try and soothe her, because if their family comes in and there’s bruises, you’re done.’ I said, ‘If she starts flailing, you chart your butt off, that that’s what happened. And you get a witness. Because I mean, we were all in there. So we all chart behind you that that’s what happened.’* **(Deanna, Nurse)** 

While expressing concern about preventing the resident from being injured during care, Deanna also revealed significant concerns about the consequences workers face should the resident’s family see that they have been bruised. Similarly, Deanna stated that she did not use formal methods of restraint because of rules preventing her from doing so, while endorsing the use of informal methods, like holding their hands down. This is indicative of an environment where neither residents nor workers feel safe, and are constrained from experiencing well-being in the space that they live and work.

Another participant, Fiona, retold how she felt pressured by family to provide care that the resident had declined, and that she believed would compromise her own safety. Thus, Fiona and other workers expressed simultaneous fear of physical harm, disciplinary action resulting from forcing treatment on a resident*, as well as* professional or family discipline (and/or complaint) from neglecting to provide treatment to a resident. The stress of negotiating care provision without compromising her physical safety and risking professional sanctions or complaints seemed to undermine compassionate and relationally focused care provision, as illustrated in the following excerpt from Fiona:

*You’re told to go in the room, if they seem agitated, you’re just told to approach later, but you got family in there that are like, ‘oh, my mom or dad needs to be changed,’ So, they don’t want them sitting in soiled diapers or whatever. If they get a pressure sore, or anything, they’re at you because now they have a sore. But if you change them, and they’re fighting you, then you’re in trouble because you should have left them alone.* **(Fiona, Care Aide)** 

In this description, Fiona conveys that there is no choice she can make where she will not suffer negative consequences, whether that represents reprimand or injury. One way that workers cope in these situations is to prioritize protecting themselves, even if that means that the resident is harmed. Preventing harm to the resident can seem almost incidental in workers’ attempts to protect themselves. Yet importantly, this phenomenon is structurally generated, rather than resulting from inadequate training or incompetence. Workers’ choices are restricted significantly by the unmitigated risks presented by the conditions of their workplace.

### Preventing moral distress: How a biomedical caring imperative shapes narratives and decisions about involuntary treatment

Workers’ accounts of decision-making around involuntary treatment emphasized the moral and ethical obligations they felt to provide bodily and medical care, despite recognizing a high risk of injury to themselves or residents. Indeed, there was little reflection on the way that residents experienced these interactions. While some staff mentioned training and philosophies that prioritized taking the time to provide more relational care, many staff struggled to prioritize preserving residents’ autonomy when it was at odds with biomedical and physical treatments.^2^ In some cases, such perspectives contradicted institutional instruction. Gloria explained their sense of unease *not* forcing personal care after a resident had declined:

*I know we have, we have had a few violent residents who we are told to walk away, and it’s hard for a number of people to walk away because how can you walk away, when they need our help? So that’s a really hard thing to remove yourself from that. And, you can be told that 50 times, but until it clicks in your mind, it still doesn’t feel best to me.* **(Gloria, Care Aide)** 

This kind of dilemma in care provision was noted by management at Facility A as something some workers had expressed to them as well. As they spent more time working within an organizational culture that problematized involuntary treatment, however, workers at facility A explained they came to feel very positively about using time to approach care respectfully and prioritize resident comfort and desires.

Likewise, despite admitting some discomfort with policies restricting the practice of forcing care, Gloria was encouraged by their employer to prioritize their own safety and to avoid risk by respecting residents’ refusal of care. This organizational norm made space for the participant to grapple with the risk of violence as a response to forced care, and the risks to the resident associated with going without care. Conversely, in an environment where some forms of involuntary treatment are normalized and expected, workers felt they had little choice but to provide care despite the risk of injury, as illustrated in this excerpt from Heidi:

*What can I do to stop myself from getting hit and kicked and punched and bit? […] Like, it’s hard to want to go to work when you know you’re gonna get kicked. And I mean, some healthcare aides refuse to do certain people, because they’re going to get kicked. Some healthcare aides refuse to do others because at one time, they were paranoid and called them the ringleader of a cult. It’s like, good for you. But at the end of the day, I can’t refuse to give them their medication because they may swing at me. I just have to take the chance and be quick enough to get out of the way. And I mean, no, I don’t think having the power to restrain them is the answer, because, at one point, I do think that was an option and obviously that was abused, and it was taken away. Right? Because we wouldn’t have a policy that says you can’t do that. So, like, where’s the happy medium? What are my rights as a staff member? Like, do I not have a right to a safe workplace, for staff and residents?* **(Heidi, Nurse)** 

Heidi conveys a sense of resignation around experiencing violence when providing care and, like many other workers, expressed that they did not feel safe doing their job because of the risk of violence. Although Heidi does not view formal restraint measures as acceptable, she does not believe that she has the option *not* to provide some care (such as administering medications). It is especially clear that Heidi does not feel supported by either co-workers or the organization itself. In contrast to other participants’ reflections on non-forced care and residents’ psychological safety, Heidi emphasizes that her sense of safety has been undermined, and does not reflect on residents’ experiences and interpretations of involuntary treatment. Instead, she feels responsible for providing care despite the risk of harm to herself, working with colleagues who opt out of potentially dangerous interactions with residents, in an already understaffed sector. Despite some resistance and rejection of involuntary care, there is also a sense of helplessness; in facing this dilemma, Heidi feels she only has one viable choice.

In the excerpt below, Mira describes a more overt employment of force in bodily care, with a similar rationale:

*With a guy who needs three people, he kicks, but the goal of care is to make sure he’s got good hygiene. Like you don’t want him sitting soiled for the whole shift, right? So you got to do what you got to do and then like make sure, you are prepared, and he has his meds, and you have the right number of staff.* ***(Mira, Nurse)*** 

Although involuntary treatment is shaped by residents’ resistance as well as by inadequate staffing levels, the normalization and acceptance of these practices allows coercion to permeate situations (like bodily care) that arguably do not call for worker intervention or use of force. Mira justified her use of force, and the risk of being injured by the resident’s resistance, by emphasizing the importance of good hygiene or cleanliness over other potential harm. This description, and others, represent institutional structures and routines that depend upon involuntary measures to provide treatment without harm to workers.

## Discussion

Our analysis of worker perceptions of the acceptability and practice of involuntary treatment in LTRC **highlights the normalization of non-consensual “care” practices within broader institutional structures that seem to function, not in spite of, but because of routinized violence.** An interesting pattern emerged wherein workers described providing some forms of involuntary treatment to residents they perceived as potentially aggressive or creating a risk to workers, and more subtle forms of coercion and non-consensual care provision with residents they viewed as less of a risk. Our findings suggest a self-perpetuating, cyclical struggle for power, where workers’ use of coercive treatment practices can escalate synergistically with residents’ resistance. This cycle erodes relationships between workers and residents, as neither are safe in their interactions. This was particularly apparent in interview data, as participants emphasized the importance of providing treatment over respecting residents’ bodily autonomy and agency, and despite risks to their own safety. Caught in this pattern, work practices and indeed institutional policies themselves inadvertently encourage residents’ resistance. Participants described structured routines of involuntary treatment, like approaching with more workers and using informal restraint as preventative measures. This reflects an institutional reliance on involuntary treatment practices, which function first to ensure that treatment is provided at all within understaffed and under-resourced environments, and second, to control residents’ resistance to prevent workers from being harmed.

Participants themselves were caught up in this cycle, noting that the escalation of force generated more resistance from residents, which meant that more forceful methods were needed to complete care. Residents developed a more defensive baseline, as they repeatedly experienced forceful and potentially traumatizing invasions of their bodily autonomy. Other research examining care workers’ perceptions of violence against residents has suggested that workers may even retaliate against residents who resist care by intensifying the use of force and restraint (Myhre et al., 2020). This pattern did emerge in our analysis, but more fundamentally, our findings reflect a broader normalization of violence as a “part of the job,” despite some ambivalence and alternative viewpoints (particularly in the non-forced care setting). Using a Foucauldian lens to examine this pattern reveals the carceral nature of the LTRC environment, particularly in relation to violence prevention, extending previous scholarship on surveillance of older adults (Mortenson et al., 2015). Institutional exercises of power over residents, by disregarding residents’ refusal of care, are followed by defensive resistance. This results in the labelling of particular residents as “violent,” using formal, structural means and more informal discursive means, like framing the resident as challenging in conversations, which can lead workers to take a more offensive approach to care.

**As such, structural conditions (i.e., norms, staffing levels, task-oriented environments) contextualize and facilitate pervasive, cyclical violence in LTRC.** In alignment with other research (Lund et al., 2024), some workers in the present study expressed significant distress and malaise with providing involuntary treatment, while others seemed more detached from relational aspects of care and stressed the importance of efficacy in bodily care and self-protection. Participants also rationalized their acceptance of involuntary treatment practices by framing residents as unable to know “what’s best” for them (especially in relation to hygiene), reflecting a paternalistic, medico-legal conceptualization of “best interest.” Moreover, participants expressed being motivated by fear of reprimand or judgment from the families of residents, which is consistent with other research indicating that relatives of residents tend to endorse coercive practices to maintain their family member’s “dignity” (Gjerberg et al., 2016). Although family advocacy is understandable in the context of resource constraints and widespread concerns of neglect in LTRC, the broader acceptability of involuntary care seems to stem from a perception that there is no better, available option.

In light of this, the practices observed in and described by participants from Facility A were particularly promising. Rather than continuing to perpetuate cyclical power struggles and escalating forceful tactics to avoid injury from residents’ resistance, workers there attempted to meet resistance with flexibility, curiosity, and attention to specific individuals in specific situations, as detailed elsewhere (Goodman et al., 2025). Worker education and person-centered care interventions alone have limited efficacy in creating sustainable improvements to LTRC conditions (Berkovic et al., 2024). Facility A’s disruption of violent and harmful treatment routines exemplifies the extent to which violence in LTRC is a structural rather than interpersonal issue, requiring change not only in institutional norms, but policy, practice, and funding levels.

Participants described the significant complexity of navigating policies surrounding involuntary treatment and restraints. While emphasizing prohibitions on physical restraints, staff still detailed routinized processes such as multiple staff physically holding residents’ hands against grab bars or dressers to prevent a resident from striking out and holding residents’ hands during care provision. These varying interpretations of what constitutes restraint reflect a lack of clarity and regulation of involuntary treatment and “informal” restraints in this sector.

### Limitations

Our analysis relies on workers’ perspectives and researchers’ interpretations of their observed actions with limited insight into residents’ experiences beyond what we could observe in public spaces of facilities (i.e., outside resident rooms) from the residents who provided written consent or proxy consent. As such, more research is needed about the experiences of involuntary treatment and issues of consent and coercion among those living with dementia in LTRC. Nonetheless, the data we obtained included situations of coercive and involuntary treatment, which was often normalized and justified. More in-depth observational research in a wider variety of LTRC facilities is also needed to explore the transferability of these findings to other contexts and settings.

## Conclusion

LTRC is often experienced as unsafe by residents and workers (Herron et al., 2024), and our findings indicate how safety-related threats and risks (including fears of physical injury and organizational, professional, and family reprimand) impact the quality of care and quality of life of people who live and work in these settings. Workers’ concern for their own safety influences perceptions of and decisions about providing involuntary treatment, which can impact residents’ safety and well-being. Manifestations of involuntary treatment go beyond regulated forms of restraint, necessitating interventions at an institutional level. These findings do *not* suggest that violence in LTRC should be attributed to individual workers or the way that they approach care provision. Structural and organizational factors, like understaffing, stringent time constraints, employment precarity, and fears of reprimand contribute to the deterioration of conditions in LTRC. As a result, worker education is important but insufficient for improving the quality of care. If workers are provided with safe and supportive conditions of labor, they are better equipped to provide compassionate, high-quality care to residents. Neither workers nor residents deserve to experience violence in LTRC. Despite the continued normalization and acceptance of some forms of violence (Brophy et al., 2018; Funk et al., 2021; Gates et al., 1999), this is unacceptable and inhibits people from enjoying a just living and work environment.

Forceful and frightening worker conduct, like having multiple workers present while physically forcing care, can be a way workers try to protect themselves from harm when they lack time or resources to provide care without force. Shifting these care practices can be particularly challenging when these practices are perceived as enhancing worker safety. Yet, forced care can actually increase workers’ risk of harm by triggering responsive or aggressive behavior in residents, reinforcing the need to use non-consensual methods to provide care. Both workers and residents are harmed by this self-perpetuating cycle. Moreover, in a context of heightened fear of one’s personal safety, workers had less regard for the safety or well-being of residents. However, when workers felt safe in their workplace, they had much greater capacity to engage in compassionate relational care and expressed more satisfaction and positive sentiment toward residents and their work. In sum, major environmental and structural changes in LTRC, including recognition and regulation of more informal forms of restraint, alongside greater understanding of the cyclical nature of force in these settings, can promote reciprocally caring relationships in these spaces.

## Data Availability

The authors do not have permission to share the data. This study was not preregistered.

## Footnotes

1 Broda is a brand name for a wheelchair that includes a seatbelt and tray, both of which can be used as physical restraints (although typically not condoned in policy).

2 Within a biomedical model of care that prioritizes medical treatment of physical bodies over more relational or comfort-oriented aspects of care (Banerjee, 2023; Reimer & Keller, 2009), giving medication and ensuring adequate nutrition and hydration become rigidly defined worker responsibilities despite physical risk to the worker and violation of resident autonomy (Hande et al., 2021).
